# Supplementary material for: The Evolution and Characterization of the RNA Interference Pathways in Lophotrochozoa
Source: Genome Biol Evol. 2024 May 7;16(5):evae098. doi: 10.1093/gbe/evae098 (PMC11114477; doi:10.1093/gbe/evae098)
Supplement: evae098_Supplementary_Data [file evae098_supplementary_data.zip › Supplementary_materials.zip!/selected_miRNAs/NoGe_selected_miRNAs/mirdeep_pdf_files/NMRB01001254.1_Notospermus_geniculatus_strain_Ushimado_scaffold1254,_whole_genome_shotgun_sequence_26726.pdf]

| | | |
|---|---|---|
| Provisional ID | : | NMRB01001254.1_Notospermus_geniculatus_strain_Ushimado_scaffold1254,_whole_genome_shotgun_sequence_26726 |
| Score total | : | 538.9 |
| Score for star read(s) | : | 3.9 |
| Score for read counts | : | 530.3 |
| Score for mfe | : | 1.6 |
| Score for randfold | : | 0 |
| Score for cons. seed | : | 3 |
| Total read count | : | 1052 |
| Mature read count | : | 828 |
| Loop read count | : | 0 |
| Star read count | : | 224 |

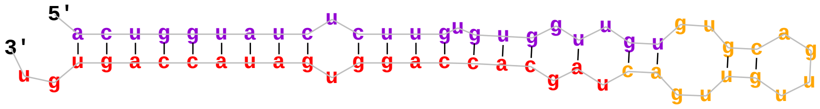


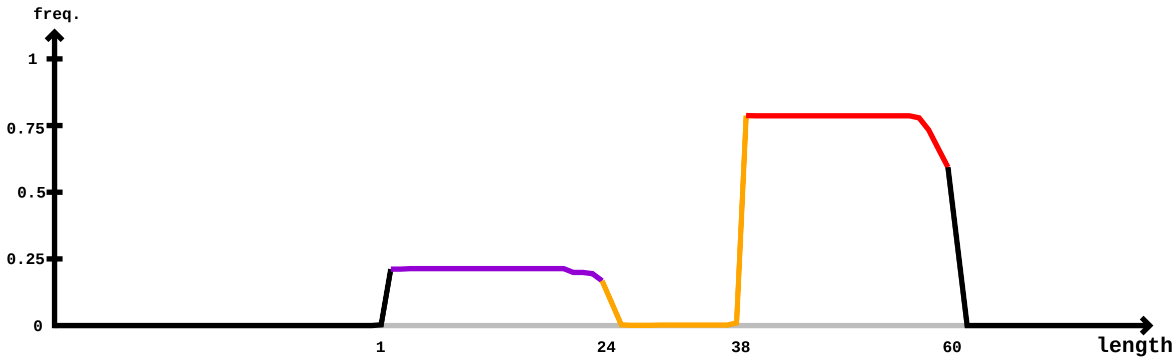


**Star** **Mature**

| | | | |
|---|---|---|---|
| 5'- gauacugugcuuuaagcuuccuccuccucacucacugguaucucuugugugguugugugcaguuguugacuagcaccaggugauaccagugugcgaguugguucaugcuaug -3' | obs | | |
| gauacugugcuuuaagcuuccuccuccucacucacugguaucucuugugugguugugugcaguuguugacuagcaccaggugauaccagugugcgaguugguucaugcuaug | exp | | |
| ..............(((.....((..(((.(.((((((((((.((((.(((.(.((..((....))..)).).))))))).)))))))))).).)))..)).....)))... | reads | mm | sample |
| .................................acugguaucucuugugugg............................................................ | 14 | 0 | Fm3 |
| .................................acugguaucucuugugugguug......................................................... | 11 | 0 | Fm3 |
| .................................acugguaucuAuugugugguugu........................................................ | 1 | 1 | Fm3 |
| .................................acugguaucucuugugugguugu........................................................ | 13 | 0 | Fm3 |
| .................................acugguaucucuugugugguuguU....................................................... | 1 | 1 | Fm3 |
| ......................................................................uagcaccaggugauaccag....................... | 44 | 0 | Fm3 |
| ......................................................................uagcaccaggugauacGag....................... | 1 | 1 | Fm3 |
| ......................................................................uagcaccaggugauacUag....................... | 1 | 1 | Fm3 |
| ......................................................................uagcaccaggugauaccagu...................... | 28 | 0 | Fm3 |
| ......................................................................uagcaccagguUauaccagu...................... | 1 | 1 | Fm3 |
| ......................................................................uagcaccaggugauacUagu...................... | 1 | 1 | Fm3 |
| ......................................................................uagcaccaggugauaccagug..................... | 39 | 0 | Fm3 |
| ......................................................................uagcaccaggugauaccagugu.................... | 119 | 0 | Fm3 |
| ......................................................................uagcaccaggugauacUagugu.................... | 1 | 1 | Fm3 |
| ......................................................................uagcaccaggugauaccUgugu.................... | 1 | 1 | Fm3 |
| ......................................................................uagcaccaggugauaccaguguU................... | 4 | 1 | Fm3 |
| | | | |
| .................................acugguaucucuugugugg............................................................ | 1 | 0 | Mm3 |
| .................................acugguaucucuugugugguu.......................................................... | 1 | 0 | Mm3 |
| .................................acugguaucucuugugugguC.......................................................... | 1 | 1 | Mm3 |
| .................................acugguaucucuugugugguugu........................................................ | 39 | 0 | Mm3 |
| .................................acugguaucucuugugugguAgu........................................................ | 1 | 1 | Mm3 |
| .................................acugguaucucuugugugguuguU....................................................... | 2 | 1 | Mm3 |
| .............................................................guuguugacuagcaccaggugauaccagugu.................... | 1 | 0 | Mm3 |
| ......................................................................uagcaccaggugauacca........................ | 1 | 0 | Mm3 |
| ......................................................................uagcaccaggugauaccagu...................... | 8 | 0 | Mm3 |
| ......................................................................uagcaccaggugauacGagu...................... | 1 | 1 | Mm3 |
| ......................................................................uagcaccaggugauaccagug..................... | 1 | 0 | Mm3 |
| ......................................................................uagcaccaggugauaccagGg..................... | 1 | 1 | Mm3 |
| ......................................................................uagcaccaggugauaccaguUu.................... | 1 | 1 | Mm3 |
| ......................................................................uagcaccaggugauaccagugC.................... | 1 | 1 | Mm3 |
| ......................................................................uagcaccaggugauaccagugG.................... | 2 | 1 | Mm3 |
| ......................................................................uagcaccaggugauaccaguAu.................... | 1 | 1 | Mm3 |

```
                                 Star                                 Mature
gauacugugcuuuaagcuuccuccuccucacucacugguaucucuugugugguugugugcaguuguugacuagcaccaggugauaccagugugcgaguugguucaugcuaug
......................................................................uagcaccaggugauaccagugu....................   26    0   Mm3
......................................................................uagcaccaggugauaccaguguU...................   1     1   Mm3

.....................................................................cuagcaccaggugauaccaguU.....................   2     1   Fm1
......................................................................uagcaccaggugauacca........................   5     0   Fm1
......................................................................uagcaccaggugauaccaU.......................   1     1   Fm1
......................................................................uagcaccaggugauaccagu......................   11    0   Fm1
......................................................................uagcaccaggugauaccagug.....................   5     0   Fm1
......................................................................uagUaccaggugauaccagugu....................   1     1   Fm1
......................................................................uagcaccaggugauaccCgugu....................   1     1   Fm1
......................................................................uagcaccaggugauaccagugG....................   15    1   Fm1
......................................................................uagcaccaggugauaccGgugu....................   1     1   Fm1
......................................................................uagcaccaggugauacUagugu....................   1     1   Fm1
......................................................................uagcaccaggugauGccagugu....................   1     1   Fm1
......................................................................uagcaccaggugauaccagugu....................   174   0   Fm1
......................................................................uagcaccaggugauaccaguguA...................   7     1   Fm1
......................................................................uagcaccaggugauaccaguguU...................   10    1   Fm1

.................................acugguaucucuugugugguugu........................................................   14    0   Fm2
.....................................................................cuagcaccaggugauaccagugG....................   1     1   Fm2
.....................................................................cuagcacAaggugauaccagugu....................   1     1   Fm2
.....................................................................cuagcaccaggugauaccagugu....................   4     0   Fm2
......................................................................uagcaccaggugauaccagug.....................   1     0   Fm2
......................................................................uagcaccaggugauaccaguU.....................   4     1   Fm2
......................................................................uagcaccGggugauaccagugu....................   1     1   Fm2
......................................................................uagcaccaggugauaccagugG....................   3     1   Fm2
......................................................................uagcaccaggugauaccagugu....................   52    0   Fm2
......................................................................Cagcaccaggugauaccagugu....................   1     1   Fm2
......................................................................uagcaccaggugauaccaguguU...................   13    1   Fm2

.................................acugguaucucuuguguUguug..........................................................   1     1   Mm1
.................................acugguaucucuugugGgguug..........................................................   1     1   Mm1
.................................acugguaucucuugugugguug..........................................................   13    0   Mm1
.................................acugguaucucuugugAgguugu........................................................   1     1   Mm1
.................................acugguaucucuuguguCguugu........................................................   1     1   Mm1
.................................acugguaucucuugugugguugu........................................................   43    0   Mm1
......................................................................uagcaccaggugauaccagu......................   11    0   Mm1
......................................................................uagcaccaggugauaccagA......................   8     1   Mm1
......................................................................uagcaccaggugauaccagug.....................   9     0   Mm1
......................................................................uagcaccaggugauacAagugu....................   1     1   Mm1
......................................................................uagcaccaggugauaccagugu....................   56    0   Mm1
......................................................................uagcaccaggGgauaccagugu....................   1     1   Mm1
......................................................................uagcGccaggugauaccagugu....................   1     1   Mm1

................................cacugguaucucuugugugguu..........................................................   3     0   Mm2
.................................acugguaucucuugugugguug..........................................................   2     0   Mm2
.................................acugguaucucuugugugguugu........................................................   54    0   Mm2
.................................acugguaucuAuugugugguugu........................................................   1     1   Mm2
.................................acugguaucucuuguguUguugu........................................................   1     1   Mm2
.................................Ncugguaucucuugugugguugu........................................................   1     1   Mm2
.................................acugguaucucuugugugguuguU.......................................................   1     1   Mm2
.................................acugguaucucuugugugguugugugcaguuguugacu.........................................   1     0   Mm2
...................................ugguaucucuugugugguugugu......................................................   2     0   Mm2
......................................................................uagcaccaggugauaccG........................   1     1   Mm2
......................................................................uagcaccaggugauacca........................   1     0   Mm2
......................................................................uagcaccaggugauaccag.......................   1     0   Mm2
......................................................................uagcaccaggugauacAagu......................   1     1   Mm2
......................................................................uagcaccaggugauaccaUu......................   1     1   Mm2
......................................................................uagcaccaggugauaccagu......................   3     0   Mm2
......................................................................uagcaccaggugauacUagug.....................   1     1   Mm2
......................................................................uagcaccaggugauaccagug.....................   10    0   Mm2
......................................................................uagcaccaggugauaccagugu....................   98    0   Mm2
......................................................................uagcaccaggugauaccagugG....................   10    1   Mm2
......................................................................uagcaccaggugauaccCgugu....................   1     1   Mm2
......................................................................uagcaccaggugauaccUgugu....................   1     1   Mm2
......................................................................Nagcaccaggugauaccagugu....................   2     1   Mm2
......................................................................uagcaccaggugauacUagugu....................   3     1   Mm2
......................................................................uagcaccaggugauaccaguguU...................   4     1   Mm2
......................................................................uagcaccaggugauaccaguguA...................   3     1   Mm2
```
